# Sectional Anatomy Quiz–IΧ

**DOI:** 10.22038/AOJNMB.2022.55248.1382

**Published:** 2023

**Authors:** Muhammad Azaan Khan, Geoffrey T Murphy, Rashid Hashmi

**Affiliations:** Rural Medical School, University of New South Wales (UNSW), Wagga Wagga, NSW, Australia

**Keywords:** Pons, Cerebellum, Sub-arachnoid haemorrhage, Epidural haemorrhage CT brain

## Abstract

This series lists a pictorial quiz pertaining to identification of normal and abnormal anatomical structures and landmarks at a given level on computed tomography (CT). Readers are expected to identify and appreciate the changes from normal anatomy and variations of a given pathology. The main structures assessed in this quiz are the pons, ventricular system of the brain, and the basal cisterns. Particular emphasis is placed on the presentations of intra-cranial haemorrhages, particularly sub-arachnoid and epidural haemorrhages, and masses around the region of the pons, midbrain and cerebellum. There is also a question pertaining to increased intracranial pressure. Differential diagnoses are also given where necessary to guide clinical practice and further learning. A Points to remember section details key clinical pearls. Furthermore, key resources have been cited as recommendations for further reading. It is anticipated that this series will enhance the understanding of sectional anatomy of the brain to aid in brain CT interpretation.

**Figure 1:**
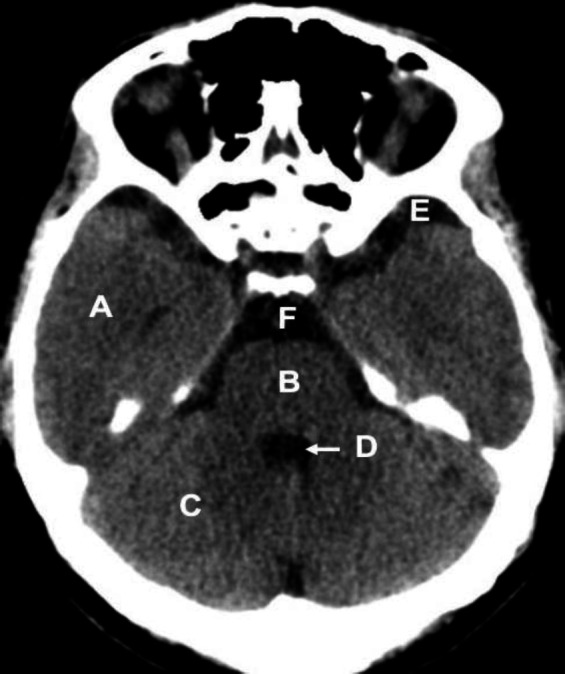
Identify the normal anatomical structures labelled **A**-**F** on a non-contrast CT brain of a middle-aged man

**Figure 2:**
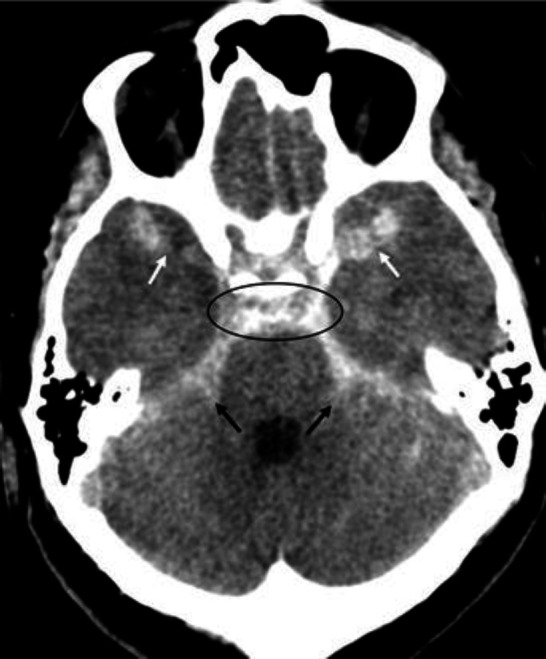
Non-contrast CT brain of a 51 year-old female with a sub-arachnoid haemorrhage. The hyperdense areas seen in the prepontine cistern (**circled**), cerebellopontine angle cistern (**black arrows**) and sylvian fissures (**white arrows**) represent acute haemorrhage

**Answers**

A: Right temporal lobe

B: Pons

C: Right cerebellar hemisphere

D: 4^th^ ventricle

E: Left sylvian fissure

F: Pre-pontine cistern

**Figure 3:**
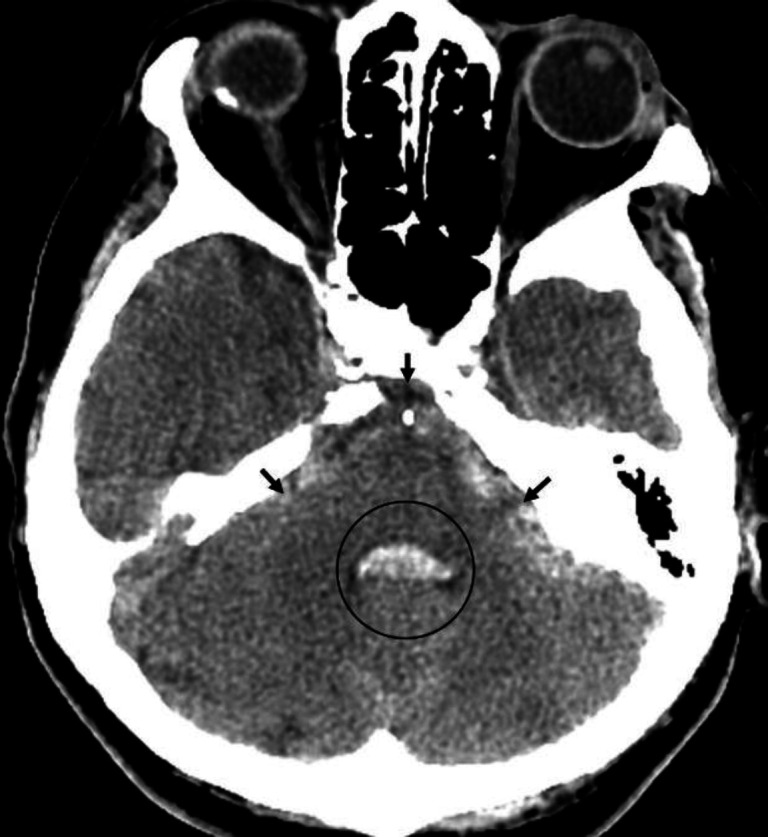
Non-contrast CT brain of a 68 year-old male with sub-arachnoid haemorrhage. Acute blood appears as a hyperdensity in the basal cisterns (**black arrows**). Note that the 4th ventricle (**black circle**) is hyperdense, indicating intraventricular extension of the haemorrhage

**Figure 4:**
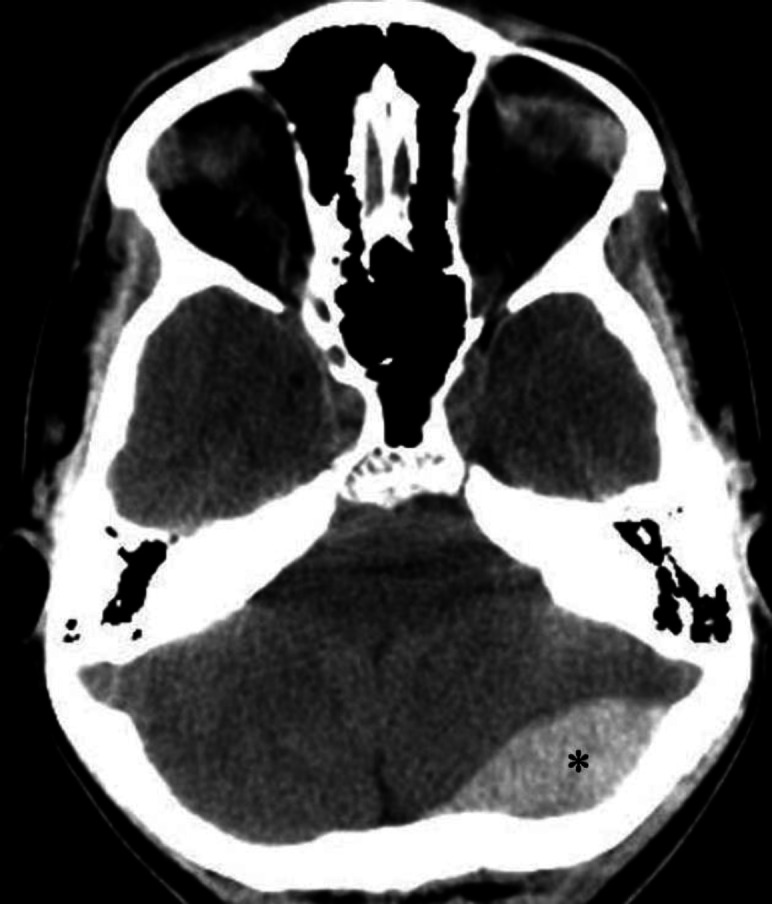
Non-contrast CT brain of an 18 year-old male with an epidural (**extra-dural**) hematoma. The hematoma (**asterisk**) appears as a sharply demarcated biconvex hyperdense area in the left occipital region. Note the mild left-to-right midline shift

**Figure 5:**
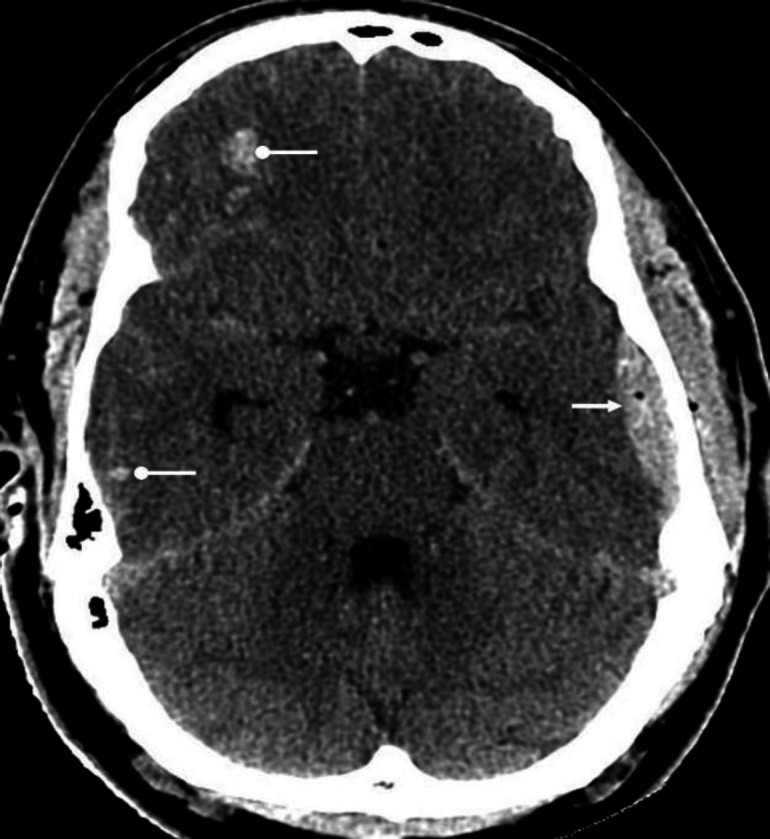
Non-contrast CT brain of a 24 year-old male following a road traffic incident. The bi-convex hyperdensity in the left temporal region (**white arrow**) is suggestive of an epidural haematoma. Note multiple foci of cerebral contusion (**white circle-head arrows**) that appear as focal hyperdense areas in the right frontal and temporal lobes

**Figure 6.**
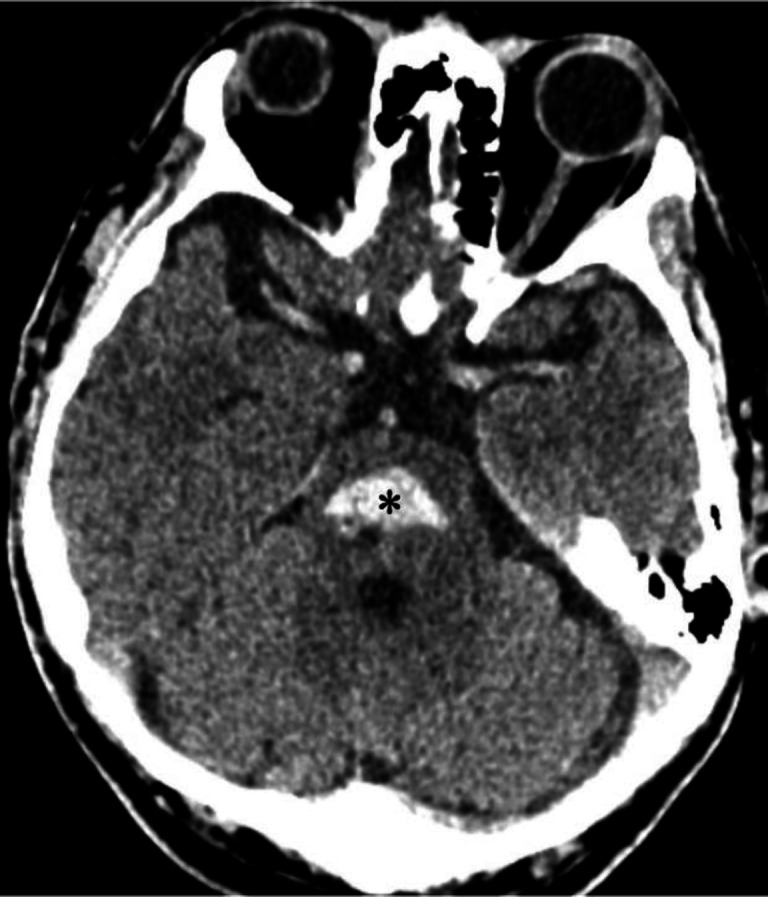
Non-contrast CT brain of a 76 year-old male with uncontrolled hypertension who was unconscious when brough to hospital after developing an acute headache. The image shows a hyperdense region centrally at the pons (**asterisk**) suggestive of a hypertensive haemorrhage

**Figure 7:**
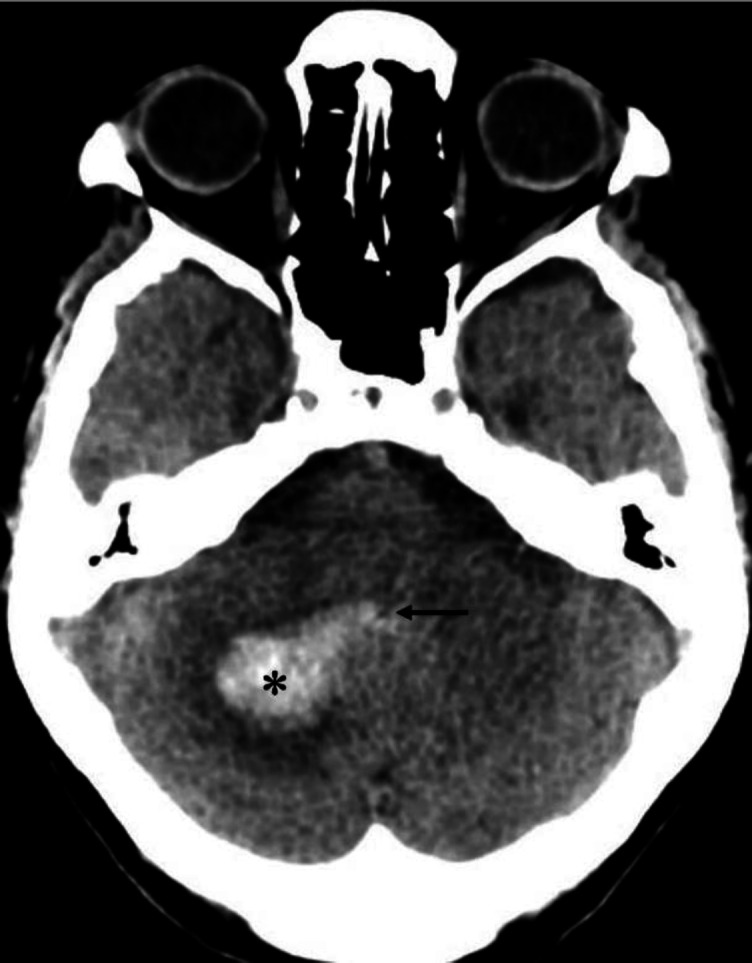
Non-contrast CT brain of a 91 year-old female with uncontrolled hypertension shows a hyperdense region in the right cerebellum (**asterisk**), corresponding to an acute haemorrhage. Note the intraventricular extension of the haemorrhage into the 4th ventricle (**black arrow**)

**Figure 8:**
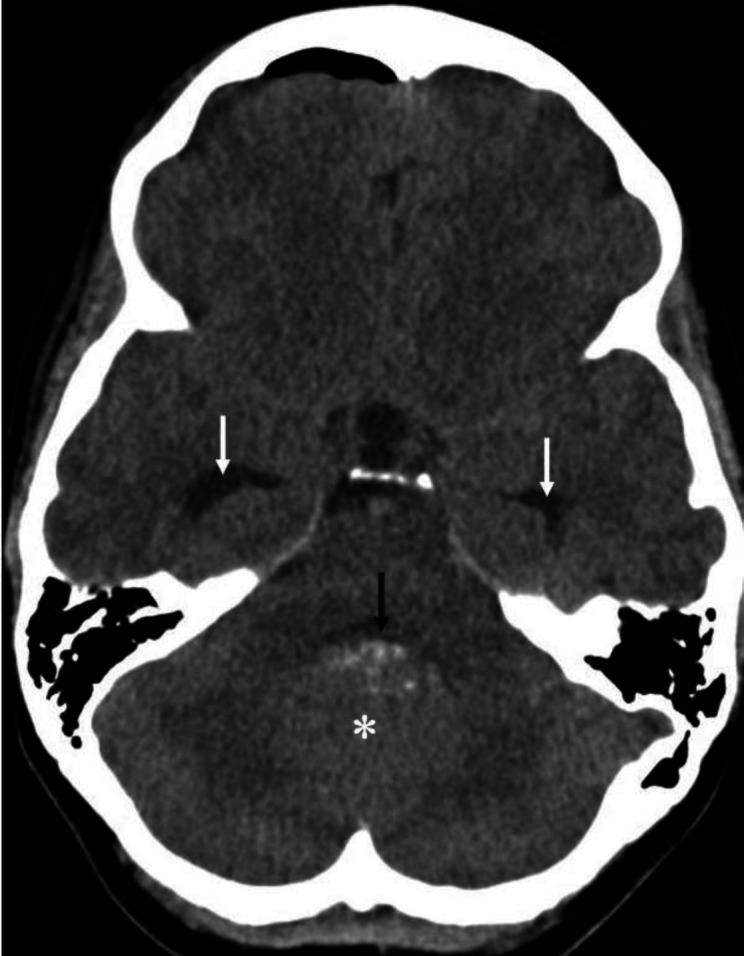
Non-contrast CT brain of a 11 year-old boy who presented with headache, vomiting and altered gait shows a slightly hyperdense midline posterior fossa mass (**asterisk**). High density areas seen in the anterior portion of the mass suggest calcification. Note the compression of the 4th ventricle (**black arrow**) by the mass. Cerebrospinal fluid hypodense areas (**white arrows**) in the bilateral temporal lobes are dilated temporal horns of the lateral ventricles and indicate increased intracranial pressure. The main differential diagnosis of a midline posterior fossa mass in a child is medulloblastoma

**Figure 9:**
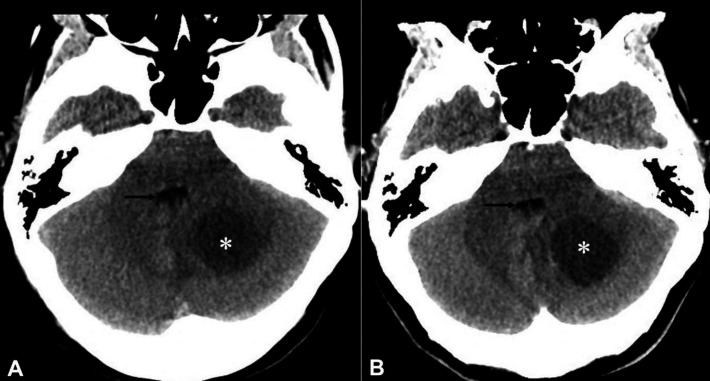
Non-contrast (**A**) and contrast enhanced (**B**) CT brain of a 24 year-old female with headache and increasing unsteadiness shows a hypodense area in the left cerebellar hemisphere (**asterisk**) with faint peripheral enhancement. Note the mass effect on the 4th ventricle (**black arrow**) by the mass. The main differential diagnosis of a low density cerebellar mass in a young adult is pilocytic astrocytoma. Other pathologies to occur in the posterior fossa of a young adult include hemangioblastoma, ependymoma, medulloblastoma, metastasis and lymphoma

**Figure 10:**
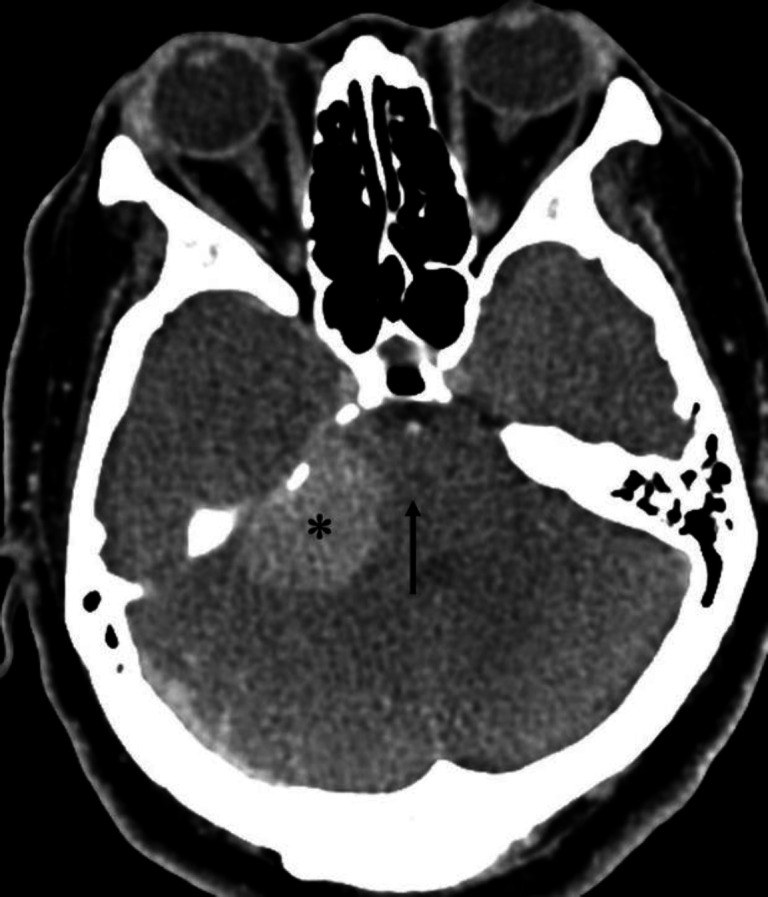
Non-contrast CT brain of a 38 year-old female shows a high density extra-axial mass in the cerebellopontine angle region (**asterisk**). Note the compression of the pons (**black arrow**) by the mass. Main differential considerations of an extra-axial mass in this location are meningioma and vestibular schwannoma. It is to be noted that distinction between an intra-axial and extra-axial location of an intracranial mass is often challenging on a single image. Hence, review of all images in different orthogonal places (axial, coronal and sagittal) is necessary for making the distinction

**Figure 11:**
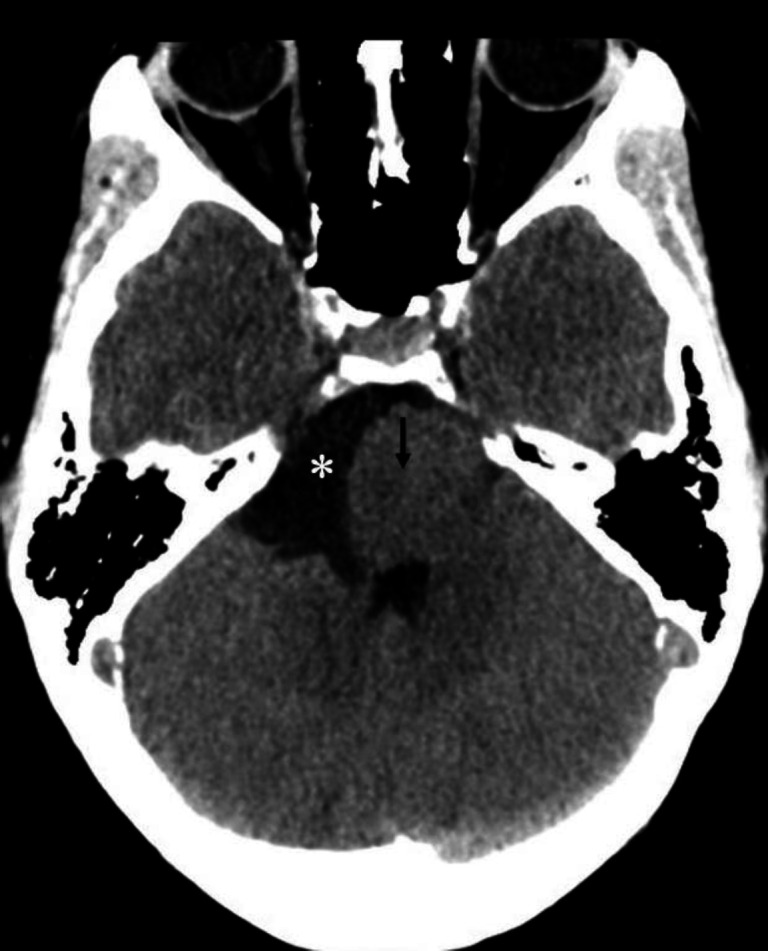
Non-contrast CT brain of a 32 year-old male shows a low density extra-axial lesion (**asterisk**) in the right cerebellopontine angle region which is compressing the pons (**black arrow**). Main differential diagnosis of a low density lesion in this location includes an epidermoid cyst and an arachnoid cyst

## Points to remember

- The pons is a midline structure spanning from below the midbrain and above the medulla. Anterior to the pons is the prepontine cistern, which contains cranial nerves V and VI, and lateral to the pons are the cerebellopontine angle cisterns, which contains cranial nerves VII and VIII. Pathology in these cisterns is usually from haemorrhage or masses which can compress the respective cranial nerves.
- The fourth ventricle is a chamber of the brain’s ventricular system which lies dorsal to the pons. It transmits CSF from the cerebral aqueduct rostrally to the obex caudally. A pontine mass may flatten the boundary between the fourth ventricle and pons (the floor of the fourth ventricle), typically a brainstem glioma in a child.
- The basal cisterns are chambers in the subarachnoid space created by a relatively greater distance between the pia and arachnoid maters, allow CSF to pool within these cisterns. Pathology is typically either the cisterns filling with blood from a subarachnoid haemorrhage or effacement of the cisterns can occur secondary to increased intracranial pressure and mass effects.

## Recommendations for Further Readings

1. Sciacca S, Lynch J, Davagnanam I, Barker R. Midbrain, Pons, and Medulla: Anatomy and Syndromes. RadioGraphics. 2019; 39(4): 1110-25.

2. Ryan S, McNicholas M, Eustace SJ. Anatomy for diagnostic imaging e-book. 3rd Ed. New York: Elsevier Health Sciences; 2011.

3. Currie S, Kennish S, Flood K. Essential Radiological Anatomy for the MRCS. Cambridge University Press. 2009.

4. Moeller T, Reif E. Pocket atlas of sectional anatomy. Computed tomography and magnetic resonance imaging. Vol. 1 Head and neck. Stuttgart: Thieme; 2013.

